# Ebola: My Head Is Full of Stories

**DOI:** 10.4269/ajtmh.14-0801

**Published:** 2015-02-04

**Authors:** Susan L. F. McLellan

**Affiliations:** Infectious Diseases Section, Tulane University School of Medicine, New Orleans, Louisiana

My head is full of stories. They play out behind my eyes like a movie reel. I suppose that is true of everyone, every day, but some stories warrant telling more than others. Perhaps especially the stories of those who did not live to tell their own tales.

My own story is simple enough. In my early 20s, I discovered that I love going to the parts of the world that others would rather avoid, and soon after that, I discovered that I love taking care of patients with diseases that others would rather not speak about. As a doctor who seeks opportunities to work in the developing world, I am often commended by friends and family for sacrificing myself, and I am a little embarrassed to point out that I do it because it is fun.

How does this work in the time of Ebola? Can it really be fun to care for Ebola patients? It certainly is not fun to be one. I have catered to cholera patients. They were not having fun either, but in a treatment center where we had competent nurses, plenty of oral rehydration solution, and an adequate supply of intravenous fluids, I could promise them that, in a week, their misery would all be over, and we would celebrate together. In the cholera ward, I could take a picture of a very sick patient with the promise that I would take another picture when they were well, and we could all laugh. This is never the case in the Ebola wards. For many, their misery would be over in a week, but the best promise I could give was that I would remember.

I volunteered to go to Kenema Government Hospital (KGH), which had opened an Ebola Treatment Unit (ETU), as a World Health Organization (WHO) short-term consultant at the end of July, when the media frenzy in the United States focused on the great peril in which the US population was being placed by the arrival of two sick missionaries from Liberia. KGH, already a clinical and research site for the care of patients with Lassa fever, was the first site in Sierra Leone to knowingly accept Ebola patients. The job description suggested that I would be a trainer and educator; the reality was that the hospital had 90 patients, 2 doctors, and a very short staff of nurses when I arrived. Granted, some of the 90 patients were not very sick yet, some were recovering, and some did not even have Ebola; however, the rest were in the grip of a very frightening disease, and what was needed was anyone willing to walk into that ward and provide care.

In the months since I have returned to the United States, I have spent many hours training potential responders: some to care for a possible patient in this country and some to deploy in one of the many ETUs springing up in affected countries. I cannot use the example of our ETU as an ideal. The theory is that anyone going in will have hours of simulated exercises and days of training in the use of personal protective equipment under his or her belt. The theory is that no one will enter the unit without a buddy and that every task will be carefully planned out. The theory is that one will know where each patient is and his or her condition before entering, and every staffer will know exactly his role. We did not quite have that luxury; it was pretty much “here's what we have to wear, here's how it goes on, here's how it comes off, there's about 85 patients in there, now let's go see what we find.” We relied on a few astoundingly competent nurses and tried to nurture the rest, many of whom were recent conscripts who walked tentatively onto the wards with panic-filled eyes and found scores of reasons to leave.

That first day seemed pretty random to me—trying to find a pattern to follow or create one in a situation where the healthcare providers had been so overwhelmed that there was little sense of organization as we responded to a chorus of “doctor, doctor!” every time that we entered the wards. I listened amazed during our break as my colleagues discussed patients by name, seeming to have kept so many people straight in their heads despite the lack of formal recordkeeping. I felt like I did not have a sense of structure, which was fair, because my colleagues had been improvising constantly as they faced rapidly rising patient loads and minimal nursing support.

Over the next day or so, my feelings changed. The names attached to faces, the faces to histories. I created my own pattern of work, adapting to what existed, working with my colleagues to develop what we needed. I admitted the first of my patients—meaning I met them in the suspect ward as the first healthcare provider to care for them—and names and stories fell into place.

I was first captured by Mariama, the quiet 6-year-old in the confirmed ward who had lost her parents and was alone in the ward. She was always on her bed, sometimes sitting, sometimes lying down, regarding the scene around her with sad eyes. It was pretty clear that she was not eating, and I knew—we all knew—that, without hydration, her chances were slim. However, she was strong enough to fight having an intravenous line placed, and the rule in the Ebola wards is that if there is any risk to a healthcare worker, do not do it. Her only chance was oral hydration, so whenever I could, I would stop and help her drink a few sips; I was gratified that, despite my bizarre appearance in personal protective equipment, she seemed willing to try for me. But I was not there very often—she was only 1 of 40+ patients in the ward with the sickest patients. Three days later, I lost Mariama. She was the first of many “if onlys”—if only we had enough staff to truly care for every patient, feed the weak, clean the messes, hang enough intravenous fluids, and hug the children. If only it were safe to do so.

“Doctor, I think that one is a corpse.” These words were often how we discovered that we had lost another victim. Usually, they came from the patient in the neighboring bed: the neighbor who yesterday had been translating for what was now a corpse, who perhaps was a friend or had become a friend, and who perhaps was wondering how long until he too was a corpse. Pronouncing death was a different animal than the procedure back home. No monitors, no alarms, no telemetry to warn; no application of the stethoscope to convey the aura of a physician's mystical knowledge. My first corpse was a baby brought into the suspect ward on my first night by her terrified mother. They were in the overflow tent, which I did not even realize existed until a patient came running to get me and led me out to find the mother and child camped on a mattress on the floor. I was pretty sure that the child was gone—no breaths, no pulse that I could find—but faced with affirming my first death on the Ebola wards, I feared to make some horrifying mistake and have the infant gasp back to life as her mother watched. This first time I found one of my colleagues to confirm. A veteran of a week, he no doubt found my insecurity ridiculous. I had already heard his stories of checking for corpses in the latrines. In the end, we both told the mother what she already knew. The next day, the test results came back—the child did not die of Ebola. Malaria, severe diarrhea, pneumonia: all the usual killers of small children in West Africa coexist with this new scourge. She was just another of that 22% of children who die before their 5th birthday.

Sadly, it did not take long for me to lose my anxiety over confirming death. The next morning, I headed into the recovery ward, which in theory, should have been populated only by convalescents. In practice, when patients were brought by ambulance in the middle of the night, they were deposited wherever there was an empty bed, to be discovered in the morning. The intended inhabitants of the recovery ward, the survivors, were a tenacious lot—battle-weary, hardened, and miraculously alive, having passed through the gates of hell and lived to tell the tale. Bit by bit, they were marshalling their strength, looking to the future, remembering what it was like to hope for more of life than just another day, while digesting the losses of friends and family. The invasion of this little community of recuperating victors by novices just beginning their journey was not always appreciated. That morning, when I asked the “president” of the ward (so assigned by my colleagues in appreciation of his assumed role of ward leader) how everyone was doing, I was informed that the new woman brought in late the previous night had been keeping them all awake with her cries, and perhaps I should check on her first. Noting the oppressive silence from that corner of the room, I approached with some trepidation. She lay on her back, hands thrown over her head, an expression of open-mouthed astonishment on her face. She would not be keeping anyone else awake. I came to know that look too well over the next few weeks. Ebola brings death in many ways, but for some, the final hours seem to be filled with demons.

I met Gabriel in the suspect ward on my second day: another young man destined to be one of “my” patients. Already 4 days into illness, he asked me hopefully between hiccoughs how long it would take to get the test results back. Looking into his bloodshot eyes as I held his tremulous hand (subconsciously searching for a vein), I thought to myself that no tests were needed; he would soon be moving into the confirmed ward. But Gabriel refused to give up hope. Despite the inevitably positive test, despite constant diarrhea, despite watching his wardmates die around him, every day Gabriel told me that he was better, his optimism belied by the fear and pleading in his eyes. He tried to eat and drink; we added intravenous fluids, antibiotics, antiemetics. One morning, though, he was speaking to no one who was present. We found him with his intravenous line torn out, blood spilt on the bed and floor, terrified. My colleague thought to offer him diazepam, what we gave when we had no more to offer. The only route, however, was intramuscular. For Gabriel, the demons had taken hold. He cowered and flinched on his bed at the approach of a garbed man with a needle. There was no safe way to come near him, and I reminded my friend that his safety came first as we backed away. We had nothing to offer but sympathy and the hope that his torture would end soon. It did. Thirty minutes later, Gabriel had found a peace beyond Valium.

Over the next 2 weeks, there were many more of “my” patients. Many recovered; some I can even claim survived because I was there: the intravenous I placed, the support I gave. They will tell their own stories, joyous ones of recovery and hope, painful ones of loss and fear, even some of laughter and foolishness. There is the doctor with the pink boots, the one who would dance for the children playing on the porch, the day shwarma and Cokes were brought for lunch as a special treat for those who could eat. I will tell those stories too.

I will speak of the needs, the hardships, the struggle to offer care while keeping safe, and the painful choices to keep safe instead of offering care. I will attest that our presence matters and that the many responders who have chosen to participate make a difference with every day and every hour that they contribute. I will stand against policies that restrict our ability to control this epidemic at the source.

And I will tell my stories. As an educator of medical and public health students, as a colleague, as a friend, as member of my community and the human race, I get to do that. I will tell the stories of those who lived and those who died, and in doing so, perhaps convey just how much fun it can be, even in the seeming worst of times, to reach across the boundaries of geography, culture, language, economics, or just luck, and connect, for a moment or a lifetime, with someone totally different and yet so much the same.

